# Supplementary material for: Drosophila beanbag (beba) encodes an insect receptor tyrosine kinase associated with reproductive niche organization
Source: G3 (Bethesda). 2026 Aug 19;16(10):jkag227. doi: 10.1093/g3journal/jkag227 (PMC13643793; doi:10.1093/g3journal/jkag227)
Supplement: Supplementary file 1 [file jkag227_supplementary_data.pdf]

Protein sequences used in Figure 6

Species	Accession Number	Protein name
Drosophila	AAN10388.3	uncharacterized protein Dmel_CG3277 [Drosophila melanogaster]
	NP_652600.1	anaplastic lymphoma kinase, isoform A [Drosophila melanogaster]
	NP_729956.1	breathless, isoform A [Drosophila melanogaster]
	NP_651349.1	cadherin 96Ca [Drosophila melanogaster]
	NP_001014474.3	discoidin domain receptor, isoform G [Drosophila melanogaster]
	NP_477341.2	doughnut on 2, isoform A [Drosophila melanogaster]
	NP_477139.1	derailed, isoform A [Drosophila melanogaster]
	NP_610835.2	derailed 2, isoform B [Drosophila melanogaster]
	NP_476758.1	epidermal growth factor receptor, isoform A [Drosophila melanogaster]
	NP_726589.2	Eph receptor tyrosine kinase, isoform A [Drosophila melanogaster]
	NP_732286.1	heartless, isoform A [Drosophila melanogaster]
	AAC47458.1	insulin receptor [Drosophila melanogaster]
	NP_477255.1	neurospecific receptor kinase [Drosophila melanogaster]
	NP_523705.2	off-track, isoform A [Drosophila melanogaster]
	NP_523509.2	PDGF- and VEGF-receptor related, isoform A [Drosophila melanogaster]
	NP_477044.1	Ret oncogene, isoform A [Drosophila melanogaster]
	NP_511114.2	sevenless [Drosophila melanogaster]
	NP_523928.1	Tie-like receptor tyrosine kinase [Drosophila melanogaster]
	NP_001097212.2	torso, isoform D [Drosophila melanogaster]
	NP_476762.1	torso, isoform A [Drosophila melanogaster]
	AAA28677.1	MAP kinase [Drosophila melanogaster]
Mouse	NP_031465.2	ALK tyrosine kinase receptor precursor [Mus musculus]
	NP_031610.1	epithelial discoidin domain-containing receptor 1 isoform 1 precursor [Mus musculus]
	NP_072075.2	discoidin domain-containing receptor 2 precursor [Mus musculus]
	NP_997538.1	epidermal growth factor receptor isoform 1 precursor [Mus musculus]
	NP_076069.2	ephrin type-A receptor 1 precursor [Mus musculus]
	NP_001003817.1	receptor tyrosine-protein kinase erbB-2 precursor [Mus musculus]
	NP_034283.1	receptor tyrosine-protein kinase erbB-3 precursor [Mus musculus]
	NP_034284.1	receptor tyrosine-protein kinase erbB-4 precursor [Mus musculus]
	NP_034336.2	fibroblast growth factor receptor 1 isoform 1 precursor [Mus musculus]
	NP_034337.2	fibroblast growth factor receptor 2 isoform IIIc [Mus musculus]
	NP_032036.2	fibroblast growth factor receptor 3 isoform 1 precursor [Mus musculus]
	NP_034698.2	insulin receptor isoform A preproprotein [Mus musculus]
	NP_976220.2	leukocyte tyrosine kinase receptor isoform D precursor [Mus musculus]
	AAP94280.1	neurotrophic tyrosine kinase receptor [Mus musculus]
	EDL37884.1	platelet derived growth factor receptor, alpha polypeptide, isoform CRA_b [Mus musculus]
	EDL09787.1	platelet derived growth factor receptor, beta polypeptide, isoform CRA_a [Mus musculus]
	NP_033076.2	proto-oncogene tyrosine-protein kinase receptor Ret isoform a precursor [Mus musculus]
	BAA75480.1	Ror1 [Mus musculus]
	NP_038874.3	tyrosine-protein kinase transmembrane receptor ROR2 precursor [Mus musculus]
	NP_035717.2	tyrosine-protein kinase receptor Tie-1 precursor [Mus musculus]
	NP_034358.2	vascular endothelial growth factor receptor 1 isoform 1 precursor [Mus musculus]
	NP_034742.2	vascular endothelial growth factor receptor 2 isoform 1 precursor [Mus musculus]

	NP_032055.1	vascular endothelial growth factor receptor 3 precursor [Mus musculus]
	NP_766479.1	NOK tyrosine-protein kinase STYK1 [Mus musculus]
	NP_001344044.1	mitogen-activated protein kinase 1 [Mus musculus]
Human	NP_004295.2	ALK tyrosine kinase receptor isoform 1 precursor [Homo sapiens]
	AQY76781.1	DDR1 - discoidin domain receptor tyrosine kinase 1
	NP_001341912.1	discoidin domain-containing receptor 2 precursor [Homo sapiens]
	AAH94761.1	EGFR protein [Homo sapiens]
	AAI30292.1	EPH receptor A1 [Homo sapiens]
	NP_004439.2	receptor tyrosine-protein kinase erbB-2 isoform a precursor [Homo sapiens]
	NP_001973.2	receptor tyrosine-protein kinase erbB-3 isoform 1 precursor [Homo sapiens]
	NP_005226.1	receptor tyrosine-protein kinase erbB-4 isoform JM-a/CVT-1 precursor [Homo sapiens]
	AAH18128.1	Fibroblast growth factor receptor 1 [Homo sapiens]
	AAA52449.1	fibroblast growth factor receptor 2 [Homo sapiens]
	AAF63380.1	fibroblast growth factor receptor 3 [Homo sapiens]
	AAI43722.1	IGF1R protein [Homo sapiens]
	AAA59452.1	INSR insulin receptor [Homo sapiens]
	NP_002335.2	leukocyte tyrosine kinase receptor isoform 1 precursor [Homo sapiens]
	NP_001336665.1	Macrophage-stimulating protein receptor isoform 1 preproprotein [Homo sapiens]
	AAA59591.1	MET proto-oncogene protein [Homo sapiens]
	AAI09100.1	muscle skeletal receptor tyrosine-protein kinase protein [Homo sapiens]
	AJM21857.1	NOK-Styk1 [Homo sapiens]
	NP_001012331.1	NTRK high affinity nerve growth factor receptor isoform 1 precursor [Homo sapiens]
	NP_002812.2	inactive tyrosine-protein kinase 7 isoform a precursor [Homo sapiens]
	NP_005003.2	inactive tyrosine-protein kinase transmembrane receptor ROR1 isoform 1 precursor [Homo sapiens]
	NP_004551.2	tyrosine-protein kinase transmembrane receptor ROR2 isoform 1 precursor [Homo sapiens]
	NP_002935.2	proto-oncogene tyrosine-protein kinase ROS precursor [Homo sapiens]
	NP_002949.2	tyrosine-protein kinase RYK isoform 2 precursor [Homo sapiens]
	EAW57022.1	TAM/AXL receptor tyrosine kinase, isoform CRA_a [Homo sapiens]
	NP_006334.2	tyrosine-protein kinase TAM/Mer precursor [Homo sapiens]
	NP_006284.2	tyrosine-protein kinase receptor TYRO3 isoform 1 precursor [Homo sapiens]
	NP_005415.1	tyrosine-protein kinase receptor Tie-1 isoform 1 precursor [Homo sapiens]
	XP_016875974.1	vascular endothelial growth factor receptor 1 isoform X1 [Homo sapiens]
	NP_002244.1	vascular endothelial growth factor receptor 2 precursor [Homo sapiens]
	XP_011532780.1	vascular endothelial growth factor receptor 3 isoform X1 [Homo sapiens]
	NP_002736.3	mitogen-activated protein kinase 1 [Homo sapiens]
	AAB97833.1	serine threonine kinase 11 [Homo sapiens]

Protein sequences used in Figure 7

Class	Order	Species	Accession Number	Protein name
Insecta	Diptera	*Drosophila simulans*	XP_016023263.1	macrophage colony-stimulating factor 1 receptor 1 [Drosophila simulans]
		Drosophila sechellia	XP_002037704.1	platelet-derived growth factor receptor beta [Drosophila sechellia]
		Drosophila yakuba	XP_002087804.2	platelet-derived growth factor receptor beta [Drosophila yakuba]
		Drosophila erecta	XP_001968541.1	platelet-derived growth factor receptor alpha [Drosophila erecta]
		Bactrocera dorsalis	XP_029406349.1	mast/stem cell growth factor receptor Kit [Bactrocera dorsalis]
		Bactrocera latifrons	XP_018784871.1	receptor-type tyrosine-protein kinase FLT3 isoform X1 [Bactrocera latifrons]
		Bactrocera latifrons	XP_018784875.1	receptor-type tyrosine-protein kinase FLT3 isoform X2 [Bactrocera latifrons]
		Rhagoletis zephyria	XP_017479635.1	mast/stem cell growth factor receptor Kit [Rhagoletis zephyria]
		Zeugodacus cucurbitae	XP_011177197.1	receptor-type tyrosine-protein kinase FLT3 [Zeugodacus cucurbitae]
		Ceratitis capitata	XP_012158328.1	fibroblast growth factor receptor 3 [Ceratitis capitata]
		Bactrocera oleae	XP_014091886.1	proto-oncogene tyrosine-protein kinase receptor Ret [Bactrocera oleae]
		Lucilia cuprina	XP_023296241.1	receptor-type tyrosine-protein kinase FLT3 [Lucilia cuprina]
		Lucilia cuprina	KNC28293.1	hypothetical protein FF38_13986, partial [Lucilia cuprina]
		Sarcophaga bullata	TMW48237.1	hypothetical protein DOY81_006679 [Sarcophaga bullata]
		Anopheles darlingi	ETN59928.1	hypothetical protein AND_008464 [Anopheles darlingi]
		Clunio marinus	CRK99865.1	CLUMA_CG013168, isoform A [Clunio marinus]
	Blattodea	*Cryptotermes secundus*	XP_023703731.1	angiopoietin-1 receptor [Cryptotermes secundus]
		Zootermopsis nevadensis	XP_021924805.1	platelet-derived growth factor receptor beta-like [Zootermopsis nevadensis]
	Lepidoptera	*Galleria mellonella*	XP_026762031.1	fibroblast growth factor receptor 3-like [Galleria mellonella]
		Amyelois-transitella	XP_013183238.1	fibroblast growth factor receptor 2 isoform X1 [Amyelois transitella]
		Spodoptera litura	XP_022814027.1	uncharacterized protein LOC111347874 [Spodoptera litura]
		Vanessa tameamea	XP_026492290.1	fibroblast growth factor receptor 3-like [Vanessa tameamea]
		Pieris rapae	XP_022117975.1	fibroblast growth factor receptor 3-like [Pieris rapae]
	Coleoptera	*Agrilus planipennis*	XP_025832128.1	platelet-derived growth factor receptor alpha isoform X1 [Agrilus planipennis]
		Agrilus planipennis	XP_025832129.1	platelet-derived growth factor receptor alpha isoform X2 [Agrilus planipennis]
Anthozoa	Sclerctinia	*Stylophora pistillata*	XP_022800499.1	uncharacterized protein LOC111338304 isoform X2 [Stylophora pistillata]
Branchiopoda	Cladocera	*Daphina magna*	KZS15239.1	Tyrosine-protein kinase STYK1 [Daphnia magna]
		Daphina magna	KZS18058.1	Uncharacterized protein APZ42_016056 [Daphnia magna]
